# Poloxamer-Based Biomaterial as a Pharmaceutical Strategy to Improve the Ivermectin Performance

**DOI:** 10.3390/pharmaceutics17091101

**Published:** 2025-08-23

**Authors:** Belén Alejandra Mezzano, Maria Soledad Bueno, Valeria Cintia Fuertes, Marcela Raquel Longhi, Claudia Garnero

**Affiliations:** 1 Departamento de Ciencias Farmacéuticas, Facultad de Ciencias Químicas, Universidad Nacional de Córdoba, Unidad de Investigación y Desarrollo en Tecnología Farmacéutica (UNITEFA) CONICET-UNC, Córdoba X5000HUA, Argentina; belen.mezzano@unc.edu.ar (B.A.M.); soledad.bueno@unc.edu.ar (M.S.B.); mrlonghi@unc.edu.ar (M.R.L.); 2 Departamento de Fisicoquímica, Facultad de Ciencias Químicas, Universidad Nacional de Córdoba, Instituto de Investigaciones en Fisicoquímica de Córdoba (INFIQC) CONICET-UNC, Córdoba X5000HUA, Argentina; vfuertes@unc.edu.ar

**Keywords:** polymer, solid dispersion, fusion method, solubility, dissolution, crystallinity

## Abstract

**Background:** Poloxamers are promising biomaterials for drug delivery applications due to their ability to enhance biopharmaceutical properties. **Methods:** This study focused on designing solid dispersions of ivermectin using poloxamer 407 by the fusion method and evaluating how variables of synthesis affect the polymer’s behavior and the resulting biopharmaceutical properties of ivermectin. Poloxamer 407 was selected based on a solubility test of preformulation studies. Initially, eight formulations were developed using different synthesis conditions, including polymer proportion, cooling gradient, and final process temperature. These were assessed by several characterization studies. Finally, saturation solubility dissolution profiles and in vitro drug release were also evaluated. **Results:** A combination of techniques confirmed the compatibility between poloxamer 407 and ivermectin in the solid dispersions. The rate of temperature in the cooling process of synthesis showed a significant impact on the polymer self-assembly, affecting their ability to entrap ivermectin. The optimized solid dispersion comprised ivermectin and poloxamer 407 in a 1:1 *w*/*w* ratio prepared by rapid cooling. This decrease in the crystallinity index and the nanometric size of particles of the solid dispersions could explain their ability to improve 1600-fold the aqueous solubility, as well as enhance the drug dissolution and in vitro drug release compared to pure ivermectin. **Conclusions:** Therefore, it follows that these poloxamer-based solid dispersions are promising alternatives to improve the bioavailability of ivermectin.

## 1. Introduction

**Figure 1:**
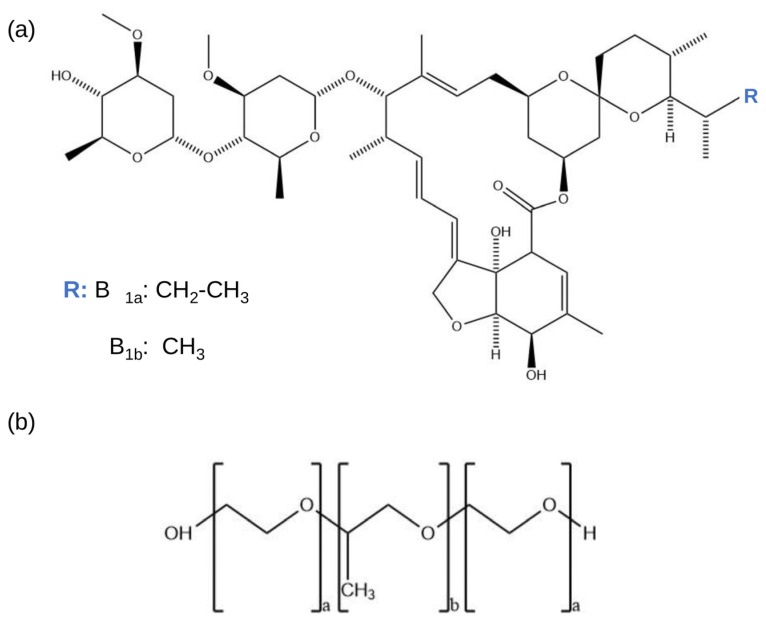
Molecular structure of (**a**) ivermectin and (**b**) poloxamer 407.

Neglected infectious diseases are a group of 14 diseases that disproportionately affect communities living in vulnerable areas, presenting themselves as an obstacle to social and economic development. However, this group of diseases can be prevented, controlled, and/or eradicated through public health measures [1]. Ivermectin (IVM) is the drug of choice to treat two types of neglected infectious diseases, Onchocerciasis and Lymphatic Filariasis [2,3]. It is an avermectin-derived semi-synthetic antiparasitic agent, consisting of an 80:20 mixture of avermectin B1a and B1b (Figure 1), which exhibits a broad spectrum of activity against ectoparasites and endoparasites [4]. Despite its wide use in both human and veterinary medicine, it belongs to class II of the biopharmaceutical classification system. In other words, this drug is characterized by good permeability, but its use is limited due to its poor aqueous solubility, which results in low oral bioavailability [5]. Various strategies have been investigated to improve the therapeutic profile of IVM [6,7].

On the other hand, advanced materials with high efficiency have attracted considerable attention for drug delivery, such as poloxamers, materials with special properties such as biocompatibility, biodegradability, and good tolerance, which have versatile applications. The U.S. Food and Drug Administration has approved several forms of poloxamer for pharmaceutical applications. This family of synthetic amphiphilic triblock copolymers (Figure 1), with a hydrophobic central block and hydrophilic lateral segments, is capable of loading water-insoluble drugs and interacting with hydrophobic surfaces and biological membranes to improve the drug availability [8,9,10]. In particular, poloxamers have been reported as suitable excipients for preparing solid dispersions (SDs) with improvements in drug wettability, solubility, and dissolution [11,12,13,14,15,16]. It can be noted that previous reports employed poloxamers as stabilizers of IVM nanocrystals, resulting in improved solubility [17,18], as well as a stabilizer of IVM-loaded lipid polymer hybrid nanoparticles, which enhance IVM solubilization and thus increase its release potential for pulmonary delivery [19]. Moreover, poloxamer 407 (P407)-based polymeric micelles incorporating IVM were investigated as an alternative to drug repositioning for the treatment of leishmaniasis [20,21].

In this study, a novel biomaterial was developed to enhance the solubility and dissolution rate of IVM. SDs are effective tools in the pharmaceutical field for enhancing drug bioavailability in both laboratory and industrial scale processes. In this investigation, the development of novel SD formulations started with the evaluation of several carriers to optimize their composition. The optimized formulations, containing P407 as the carrier, were prepared at two drug-to-polymer weight ratios of 1:1 and 1:2 by the fusion method under different cooling conditions. The resulting SDs were characterized to evaluate IVM–polymer interactions, particle size, morphology, formulation stability, and their impact on IVM solubility, dissolution, and release profile. The results demonstrated that the solubility, dissolution rate, and release of the drug were significantly enhanced by the 1:1 SDs produced through the rapid cooling of the melted sample.

## 2. Materials and Methods

### 2.1. Chemicals and Reagents

IVM was supplied by Todo Droga (Córdoba, Argentina), P407 and poloxamer 188 (P188) were sourced from BASF (Ludwigshafen, Alemania), polyvinylpyrrolidone k90 (PVP-k90) was obtained from Sigma-Aldrich (Burlington, MA, USA), both polyvinylpyrrolidone k30 (PVPk-30) and sorbitol were acquired from Pura Química (Córdoba, Argentina), polyethylene glycol 8000 (PEG8000) was provided by Fluka (Seelze, Germany), and polyethylene glycol 6000 (PEG6000) was sourced from Taurus (Buenos Aires, Argentina). All reagents were of analytical grade. Methanol was obtained from Cicarelli (Buenos Aires, Argentina). Additionally, doubly distilled and deionized water was generated using a Millipore Milli-Q water purification system (Millipore, Bedford, MA, USA). HPLC-grade solvents were used, including methanol from Sintorgan (Buenos Aires, Argentina) and acetonitrile supplied by J.T. Baker.

### 2.2. Preformulation Studies

Several excipients were evaluated for IVM solubility enhancement. An excess of IVM was added with 5 mL of aqueous solutions containing concentrations of 1%, 2%, and 5% *w*/*v* of the polymers P407, P188, PVP-k90, PVP-k30, PEG800, PEG6000, and sorbitol. The suspensions obtained were placed in a water thermostatic bath at 37.0 ± 0.1 °C (Lauda thermostatic bath) for 72 h, and sonicated twice every 24 h (Elmasonic S40 ultrasound). Then samples were then filtered through 0.45 μm pore size nylon membranes obtained from GVS (Sanford, ME, USA). Finally, the solutions were analyzed by UV–vis spectrophotometry (Agilent Cary 60 UV-visible spectrophotometer, Agilent Technologies, Santa Clara, CA, USA) at 246 nm. Each assay was performed in triplicate.

### 2.3. Solid Samples Preparation

#### 2.3.1. Solid Dispersions by the Fusion Method

P407 was selected as the optimum excipient based on the previous preformulation studies. Several SDs containing IVM and P407 were prepared using different synthesis conditions. Mixtures of IVM and P407 in 1:1 and 1:2 *w*/*w* ratios were heated at 63 °C in a beaker with continuous stirring until the carrier melted to form a homogeneous dispersion. The dispersions were solidified at two final temperatures: 0 °C and 8.4 °C, using the following cooling process conditions: rapid (without a temperature gradient), intermediate (with a temperature gradient of 18 °C every 45 min), and slow (at a rate of 6 °C every 60 min). The resulting SDs were then stored in a desiccator for 48 h, and the dried SDs were pulverized using a mortar and pestle. The powders were passed through sieves with mesh sizes of 150 and 106 µm to collect the samples retained between the two, which were then stored in a desiccator at room temperature until use.

#### 2.3.2. Physical Mixtures

The physical mixtures of IVM and P407 in the ratios of 1:1 *w*/*w* (PM1:1) and 1:2 *w*/*w* (PM1:2) were prepared by uniformly mixing the components with a mortar and pestle for 5 min.

### 2.4. Determination of Drug Content

The IVM content of the SDs was analyzed. Powdered samples (approximately 10 mg) were accurately weighed and dissolved in 25 mL of methanol:water solution (40:60 *v*/*v*). The solution was appropriately diluted with water, filtered through a 0.45 µm membrane filter (GVS, USA), and the IVM concentration was determined at 246 nm using a UV–visible spectrophotometer (Agilent Cary 60 UV–visible spectrophotometer).

### 2.5. Solid Dispersions Characterization

These studies employed several characterization techniques, including Fourier-transform infrared spectroscopy (FT-IR), X-ray powder diffraction (XRPD), scanning electron microscopy (SEM), transmission electron microscopy (TEM), differential scanning calorimetry (DSC), thermogravimetric analysis (TGA), hot stage microscopy, and dynamic light scattering (DLS).

#### 2.5.1. FT-IR Technique

FT-IR spectra were recorded on a Cary 630 FTIR Spectrophotometer equipped with a single bounce attenuated total reflectance (ATR) system (Santa Clara, CA, USA) available at UNITEFA (Unidad de Investigación y Desarrollo en Tecnología Farmacéutica, CONICET–UNC, National University of Córdoba, Córdoba, Argentina). For each sample, the spectrum was recorded in the range of 4000–650 cm^−1^, accumulating an average of 40 scans with a 4 cm^−1^ resolution. The spectra were processed with the OMNIC 8.0 software.

#### 2.5.2. XRPD Technique

The samples were measured using a Panalytical X’Pert Pro diffractometer with Bragg-Brentano geometry (at the Laboratorio de Rayos X, INFIQC, of the National University of Córdoba, Argentina), operated at 40 kV, 40 mA, Cu Kα radiation (λ = 1.5418 Å). The samples were scanned at ambient temperature from 4° to 35° in 2θ at a scan rate of 29.9 s/step and a scanning step size of 0.026°. To determine the relative degree of crystallinity of the samples, the crystallinity index (CI) was calculated using the area method based on the XRPD pattern, according to the following equation:

CI = (A_cr_/A_sample_) × 100 (1)

where A_cr_ is the area of the crystalline peaks and A_sample_ is the total area under the diffraction pattern, including both crystalline and amorphous contributions [22]. For the analysis, two characteristic crystalline peaks of pure IVM, located at 2θ: 11.25° and 13.18°, and the P407 peaks at 19.2° and 23.4°, were selected. This approach provides a relative measure of the crystalline content, similar to that used by Mansour et al. [23].

In addition, the crystallite size was calculated using the Scherrer equation:

(2) $$\tau =\;\frac{K\;\lambda }{\beta \;cos\theta }$$

where τ is the mean size of the crystalline domains, which may be smaller or equal to the grain size; *K* is a dimensionless shape factor, with a value close to unity; λ is the X-ray wavelength; β is the line broadening at half the maximum intensity (FWHM) after subtracting the instrumental line broadening (in radians); and θ is the Bragg angle [24].

#### 2.5.3. SEM Technique

The surface morphology of samples was examined using a Carl Zeiss Σigma^®^ scanning electron microscope (SEM) (at the Laboratorio de Microscopía y Análisis por Rayos X, LAMARX, of the National University of Córdoba, Argentina). A small amount of sample was sprinkled onto a double-sided carbon tape mounted on an aluminum stub, and gold vacuum metallized using a Quorum 150 sputter coater to improve the conductivity.

#### 2.5.4. TEM Technique

The morphology and particle distribution were observed by TEM using a Jeol 1200 EXII 80 KV microscope (Jeol, Tokio, Japan), located at the Laboratorio de Microscopía, Centro de Investigaciones Agropecuarias, INTA-CONICET, Córdoba, Argentina. Samples were prepared by the direct dissolution method; therefore, diluted suspensions were seeded on a sample carrier grid and stained with uranyl acetate (2%, *w*/*v*) solution. The excess uranyl was then removed, and the samples were air-dried at room temperature. The samples were subsequently examined at 80 kV. Average nanoparticle sizes were determined using ImageJ software (version 1.54g, National Institutes of Health, USA).

#### 2.5.5. Thermal Analysis Techniques

The DSC and TGA measurements were recorded on a DSC Discovery series and a TGA Discovery series (TA Instruments Inc., New Castle, DE, USA), respectively, available at UNITEFA (Unidad de Investigación y Desarrollo en Tecnología Farmacéutica, CONICET–UNC, National University of Córdoba, Córdoba, Argentina). The samples were heated at 10 °C/min under a nitrogen gas atmosphere (99.99% purity, flow rate of 50 mL/min). For DSC measurements, pinhole hermetic aluminum pans were used. The DSC and TG temperature axes were calibrated with indium (99.99% purity, mp 156.598 °C) and the Curie point (358.2 °C), respectively. Data were treated with TRIOS software (vesion5.1.1., TA Instruments Inc.).

#### 2.5.6. Hot Stage Microscopy

The physical and morphological changes to the samples that occurred during heating were observed through a polarizing microscope (Olympus BX51, Olympus Corp.,Tokio, Japan) equipped with a Linkam LTS420 heating stage (Linkam Scientific Instruments, Salfords, UK), using a 10× objective and crossed polars. Samples were heated at a constant rate of 10 °C/min. In order to provide experimental conditions similar to those of the DSC measurements, the samples were not embedded in silicone oil.

#### 2.5.7. DLS Technique

The particle size, polydispersity index (PDI), and Zeta potential (ZP) of SDs were measured using a ZetasizerNano ZS90 (Malvern Instruments, Malvern, UK), available at UNITEFA (Unidad de Investigación y Desarrollo en Tecnología Farmacéutica, CONICET–UNC, National University of Córdoba, Córdoba, Argentina). Prior to measurement, 10 mg of each sample was resuspended in 3 mL of Milli-Q water to adjust the signal level. These suspensions were shaken to ensure homogeneity before analysis. Measurements were performed at a 173° scattering angle, with 3 cycles of 70 measurements at 25 °C.

### 2.6. Solubility Studies

The effect of SDs on the saturation solubility of IVM was evaluated. An excess of IVM, PM, or SDs sufficient to reach a P407 concentration of 1.5% *w*/*v* was dispersed in 5 mL of aqueous solution and simulated gastric fluid (SGF) prepared according to USP [25]. The tubes were maintained in a water thermostatic bath (Lauda thermostatic bath) at 37.0 ± 0.1 °C for 72 h and sonicated daily (Elmasonic S40 ultrasound). After that, the samples were centrifuged at 6000 rpm for 45 min (CAPP CR-68X, Nordhausen, Germany) to remove the excess solid. Finally, the supernatant was collected, appropriately diluted, and analyzed by UV–visible spectrophotometry (Agilent Cary 60 UV–visible spectrophotometer) at 246 nm to determine the IVM concentration. Each assay was performed in triplicate.

### 2.7. Dissolution Study

The dissolution profiles of IVM, SDs, and PMs were determined using the dissolution apparatus I (SOTAX AT 7 Smart, Westborough, MA, USA). Appropriate amounts of each powder containing approximately 12 mg of IVM were placed in soft gelatin capsules. Experiments were performed in 500 mL of degassed SGF at 37.0 ± 0.5 °C and stirred at 50 rpm. Aliquots of 4 mL were taken at appropriate time intervals, replacing the extracted volume with fresh medium maintained at the same temperature. The dissolved IVM was analyzed by UV–visible spectrophotometry at 246 nm (Agilent Cary 60 UV–visible spectrophotometer). The tests were performed in triplicate. The cumulative percentages of IVM released from the capsules were calculated and expressed as the mean percentage of IVM released to the medium at each sampling time.

Additionally, dissolution profiles were evaluated using the similarity factor (*f*2), a metric to determine the proximity between in vitro dissolution profiles, calculated as:

(3) $$f2=50\log\{{\left[1+\frac{1}{n}\sum_{t=1}^{n}{\left(R_{t}-T_{t}\right)}^{2}\right]}^{-0.5}100\}$$

where *R_t_* and *T_t_* are the percentages dissolved of the reference and the test product, respectively, at each time point *t*, and *n* is the number of sampling points. When the *f*2 value is 50 or greater, the profiles are considered similar [26].

### 2.8. In Vitro Release Profile

#### 2.8.1. Dialysis Study

In vitro release profiles of SDs were evaluated utilizing the diffusion method within a dialysis bag. The samples containing 5 mg of IVM were resuspended in 5 mL of phosphate buffer pH 7.4 (PBS) and placed into a dialysis tubing cellulose membrane with a molecular weight cut-off of 14,000 Da (Sigma-Aldrich, St. Louis, MO, USA). The sealed dialysis bags were immersed in 80 mL of dialysis medium consisting of PBS:ethanol (80:20, *v*/*v*). The assay was performed at 37 °C under continuous agitation at 100 rpm. At predetermined time intervals, 1 mL of medium was withdrawn and replaced with fresh medium to maintain a constant volume. The diffusion surface area was kept constant (24 cm^2^) by using membranes of identical length and width for all experiments. The in vitro release behavior of SDs was simultaneously compared to that of free IVM (1 mg/mL). The assays were performed in triplicate, maintaining the sink condition. Prior to use, the dialysis membranes were immersed in water overnight. The IVM concentrations were measured by HPLC as described below. The release mechanism of IVM was evaluated by fitting the experimental data using the following kinetic models [27]:

As can be seen from the equations in Table 1, Q is the percentage of drug dissolved at time *t*. *k*_0_, *k*_1_, and *k_H_* are the rate constants of the zero-order, first-order, and Higuchi models, respectively, and *k_P_* is a constant that incorporates structural and geometric characteristics of the system. The Korsmeyer–Peppas model, in turn, uses the value of *n*, which is the exponent of release, to characterize the release mechanism.

The zero-order, first-order, and Higuchi models are often used to describe how drugs are released from poloxamer systems, while the Korsmeyer–Peppas model uses a power law equation to show how the drug is released from a polymeric system [28,29]. The DD Solver plugin was used to fit the equations and obtain the highest adjusted coefficient of determination (adjusted r^2^). Adjusted r^2^ was used instead of r^2^ because the models evaluated had different numbers of parameters, and the adjusted R^2^ takes this variability into account, thus facilitating a more accurate comparison between them [30].

#### 2.8.2. Chromatographic Method

An HPLC-UV procedure under isocratic conditions [31,32] was optimized and validated in accordance with standard guidelines [33]. The HPLC system was an Agilent 1100 (Agilent Technologies, Waldbronn, Germany) equipped with a column Gemini C18 150 mm 4.6 mm i.d. filled with 5 μm particles and a precolumn (guard cartridge SecurityGuard C18 4 mm 3.0 mm i.d.) supplied by Phenomenex (Phenomenex Inc., Torrance, CA, USA). To avoid interference from degradation products, the UV detection was performed at a wavelength of 245 nm. The mobile phase was acetonitrile–methanol–water (60:35:5, *v*/*v*/*v*), and a flow rate of 1 mL/min was used. The column temperature was 30 °C and the injection volume was 100 μL. The results obtained are reported as the mean of the three determinations of samples prepared in duplicate.

### 2.9. Statistical Analysis

The data were processed and graphed using Origin Pro 8.5 (OriginLab Corporation, Northampton, MA, USA). Results are presented as means and their standard deviations.

## 3. Results and Discussion

### 3.1. Preformulation of Solid Dispersions

The selection of appropriate excipients is critical for optimizing drug formulation. This study evaluated the effects of different polymeric excipients, including P407, P188, PVP-k90, PVP-k30, PEG8000, PEG6000, and sorbitol, on IVM solubility at different concentrations, as shown in Table 2.

The screening results demonstrated that all excipients enhanced the IVM solubility. However, variations in solubility behaviors were observed, evidencing the differential solubilizing capacities of each excipient and underscoring the importance of excipient selection in improving IVM solubility. Notably, P407 showed the most pronounced effect, increasing IVM solubility by more than 800-fold compared to the pure drug. It was followed by PVPk-30, P188, and PVPk-90, respectively. Therefore, due to its superior solubilizing capacity, P407 was selected as the most suitable excipient for the development of SDs.

### 3.2. Preparation of Solid Dispersions: Screening of Conditions

The fusion method was chosen for preparing the SDs to avoid using solvents, since the polymer provides a molten medium for solubilizing or dispersing the drug. Additionally, the selected polymer with a low glass transition temperature (Tg) is desirable for SD production at lower temperatures, thereby minimizing the risk of drug degradation [15,34]. The influence of different manufacturing factors, including the component ratio, cooling gradient, and final temperature of the preparation process, as summarized in Table 3, was evaluated to study the effect of the SDs on the drug properties, such as solubility and dissolution. Table 3 also presents the total drug content determined for the SDs.

### 3.3. Solid Dispersions Characterization in Solid State

The SDs were subsequently characterized and compared with PMs and the individual components using several techniques that are described in the following subsections.

#### 3.3.1. FT-IR

**Figure 2:**
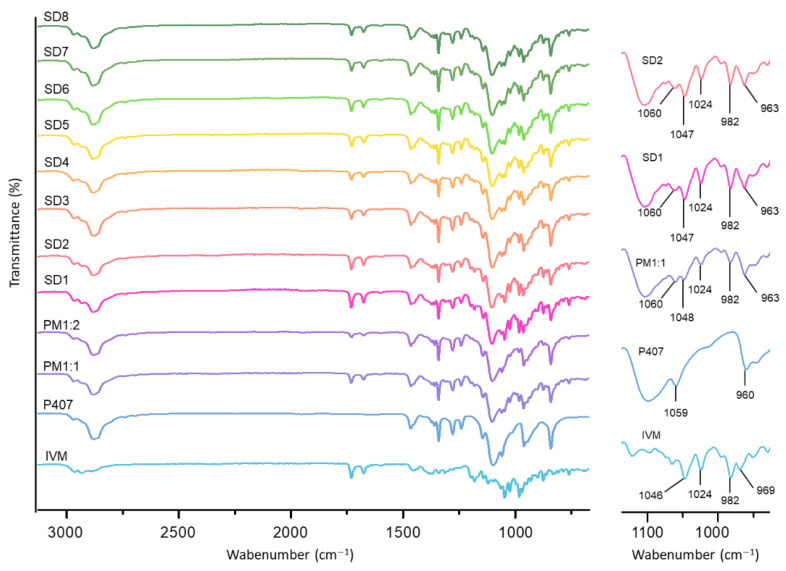
FT-IR spectra of the individual components, PMs, and SDs. A detailed view of the 1100–950 cm^−1^ region is provided on the right, where the main bands are identified.

In order to identify potential drug–excipient interactions in the formulations, FT-IR was performed. The spectra of the individual components, PMs and SDs, are shown in Figure 2. The FT-IR spectrum of IVM showed characteristic peaks at 3482 cm^−1^ (O-H stretching), 2965 cm^−1^ (C-H stretching of methyl groups), 2936 cm^−1^ (C-H axial deformation of methyl groups), 1731 cm^−1^ (C=O stretching of saturated aliphatic ketone), 1676 cm^−1^ (double bond adjacent to the -O- group in unsaturated lactones), in the range of 1382–1314 cm^−1^ (C=C stretching of ketones), and in the range of 1182–965 cm^−1^ (C-O-C stretching of aliphatic ethers), consistent with previous reports [19,35,36]. Furthermore, P407 exhibited characteristic absorption bands at 2970–2878 cm^−1^ (C-H bond vibrations), 1342 cm^−1^ (in-plane O-H bending), and 1100 cm^−1^ (C-O stretching), in agreement with the literature data [37,38]. When analyzing the spectra of the SDs and PMs, no significant changes in the positions and intensities of these characteristic peaks were observed, indicating good compatibility between the drug and the excipient, as well as the absence of substantial chemical interactions among the components of the samples. Furthermore, the spectra of PM1:2, SD3, and SD4 showed the characteristic absorption bands of IVM with lower intensity, likely due to the higher proportion of P407 in these samples.

#### 3.3.2. XRPD

**Figure 3:**
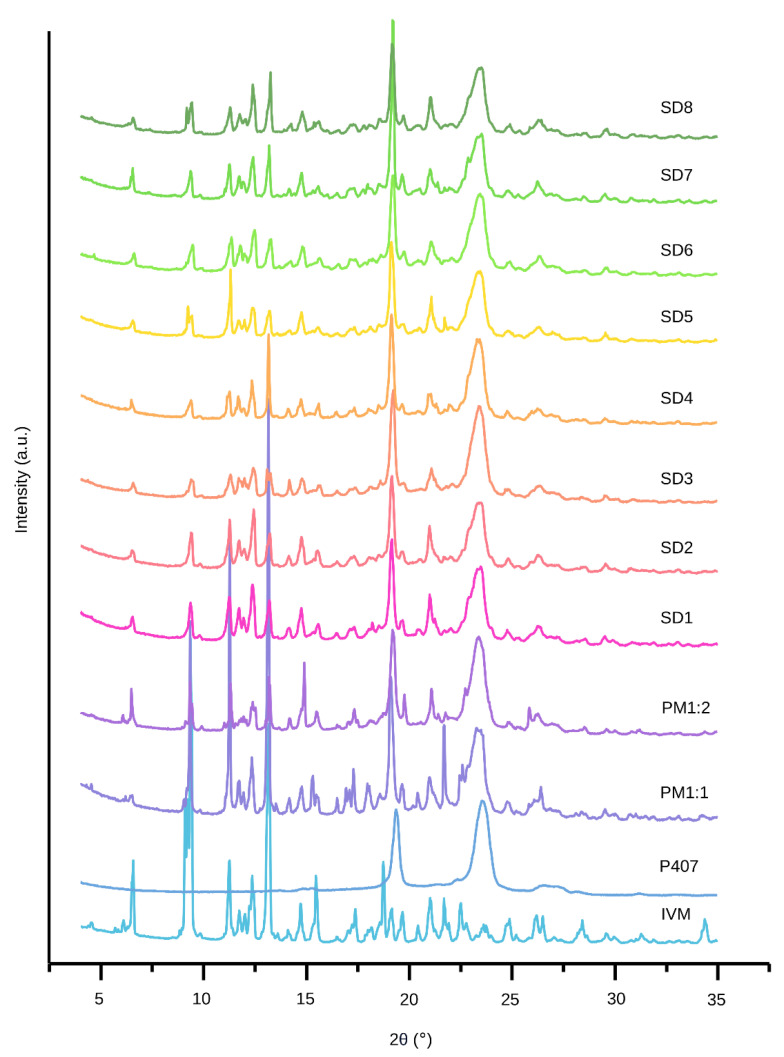
XRPD patterns of IVM, P407, PMs, and SDs.

In order to determine the physical state (crystalline or amorphous) of the powders, XRPD analysis was performed. The resulting patterns are presented in Figure 3.

The diffractograms of IVM and P407 showed good agreement with previously reported data. IVM showed characteristic peaks at 2θ: 4.55°, 6.56°, 9.38°, 11.25°, 11.74°, 12.37°, 13.18°, 14.72°, 18.74°, and 21.03°, which are indicative of its crystalline nature [35,39]. P407 showed broad diffraction peaks at 19.2° and 23.4°, which remained unchanged in SDs, suggesting the absence of new crystalline structures formed during the melting and subsequent solidification steps [13,40]. On the other hand, the diffraction patterns of both SDs and PMs are similar to the sum of the individual components, indicating no amorphization of the samples or reactions between the components. In fact, it can be noted that the crystallite size for both IVM and P407 is practically independent of the preparation method, SDs, or PMs (Table 4). As expected, the SD patterns showed the characteristic drug peaks with lower intensities and a remarkable reduction in the IVM crystallinity index (Table 4). Similar results were reported for SDs of albendazole [13] and triclabendazole [40] prepared with P407 using the fusion method. The analyzed peaks of P407 exhibited the same tendencies in crystallinity. This partial decrease in the crystallinity of both IVM and P407 contributes to enhanced solubility and an improved dissolution profile, in agreement with previous reports [13,41].

#### 3.3.3. SEM

**Figure 4:**
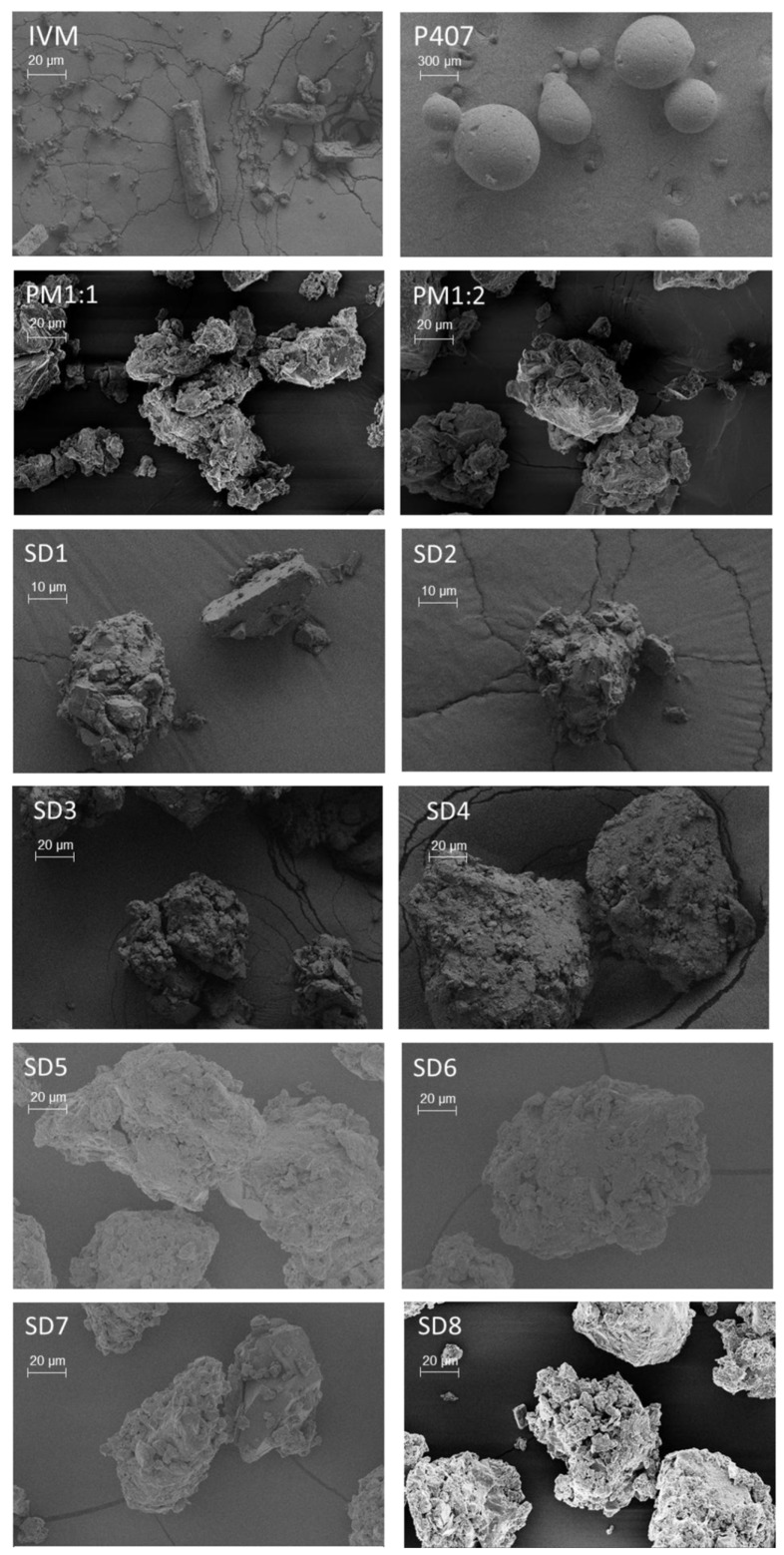
SEM images of IVM, P407, PMs, and SDs.

Subsequently, to investigate the surface morphology of the solid samples, SEM images were obtained (Figure 4).

It can be noted that IVM exhibited rod-shaped crystals of variable dimensions, with regular and smooth surfaces, while P407 presented large and smooth spherical particles. In contrast, the images of PM1:1 and PM1:2 showed irregularly shaped structures, suggesting that the individual components were fragmented during manual mixing in a mortar. The SEM images corresponding to the SDs showed homogeneous aggregated clusters with irregular edges, devoid of any characteristic IVM crystals, suggesting that the drug was dispersed in the carrier. Similar morphological features were observed regardless of the polymer content, cooling rate, or final temperature; however, SD1 and SD2 exhibited comparatively smaller particle sizes.

#### 3.3.4. Thermal Analysis

Based on FT-IR, XRPD, and SEM analyses, which did not reveal significant differences among the formulations, SD1 and SD2 were selected as model systems for further thermal studies to assess the effect of the final processing temperature used during SD preparation.

**Figure 5:**
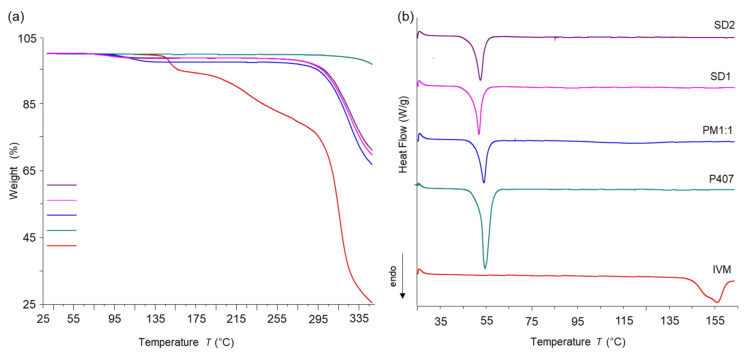
(**a**) TGA curves and (**b**) DSC curves of IVM, P407, PM1:1, SD1, and SD2.

DSC and TGA analyses of IVM, P407, PM1:1, and the selected SDs systems (SD1 and SD2) were then performed to investigate the thermal behavior of the samples (Figure 5).

The DSC curve of IVM showed an endothermic melting peak at 160.6 °C (ΔH = 79.4 J/g), accompanied by a mass loss of 4.7% in the 148–155 °C range in the TGA profile, suggesting the onset of gradual decomposition upon melting. The TGA profile of IVM was consistent with previously reported data, exhibiting thermal degradation in three stages [42,43]. The first mass loss, occurring around 150 °C, was attributed to water desorption and alteration of the crystalline form. The second mass loss, 17.3%, occurred in the 236–290 °C range and was associated with degradation of the amphiphilic esters within the molecule. This was followed by a third mass loss of 46.9%, observed over the range 309–325 °C, attributed to degradation by axial deformation of the O-H bonds and methyl groups, as reported by Callegaro Velho et al. [42]. In addition, P407 shows an endothermic event at 55.8 °C with ΔH = 132.07 J/g. This event is attributed to its melting temperature, which occurred without any associated mass loss, as confirmed by the literature data [44,45,46].

Analyzing the DSC curves of PM1:1, SD1, and SD2, the endothermic peak corresponding to P407 was observed at 55 °C (ΔH = 60.0 J/g), 53 °C (ΔH = 66.3 J/g), and 53.5 °C (ΔH = 68.0 J/g), respectively. In contrast, the endotherm associated with the melting event of IVM disappeared, presumably due to a transformation of the drug from a crystalline to an amorphous state and/or to its dissolution in the molten carrier. However, the similarity in the DSC behavior of PM1:1, SD1, and SD2 suggests that the drug could dissolve into the molten carrier during the DSC scan. To investigate this point, XRPD results were analyzed, revealing a reduction in crystallinity but not complete amorphization of the drug or the carrier in the samples. Additionally, the endothermic event attributed to P407 in the SD1 and SD2 curves exhibited a reduction in melting enthalpy and a small shift to lower temperatures, which can be explained by a low degree of IVM solubilization in the molten P407 matrix, a phenomenon previously reported by Medarevic et al. for carbamazepine–poloxamer SD [47], by Barzegar-Jalali et al. in the characterization of piroxicam SD [48], and by López Vidal et al. [18], who used P188 as a stabilizing agent for IVM nanocrystals. Therefore, these results support the formation of a molecular dispersion of IVM in the SD systems, with uniform drug distribution within the carrier. It is important to note that this process did not induce chemical or physical modifications in the components, confirming the compatibility between IVM and the selected excipient. Similar findings were reported by Callegaro Velho et al. [42], who loaded IVM in mesoporous silica particles, and by López Vidal et al., who developed IVM nanocrystals [18].

According to the TGA curves, IVM began to lose mass at around 150 °C. In contrast, the PM1:1, SD1, and SD2 exhibited significant mass loss upon heating to nearly 300 °C, evidencing that these solids were thermally more stable than untreated IVM. Additionally, the residual mass at 350 °C was 25.5, 66.7, 69.6, and 71.2% for IVM, PM1:1, SD1, and SD2, respectively. These findings suggest that IVM is stabilized by forming SD systems within the tested temperature range.

**Figure 6:**
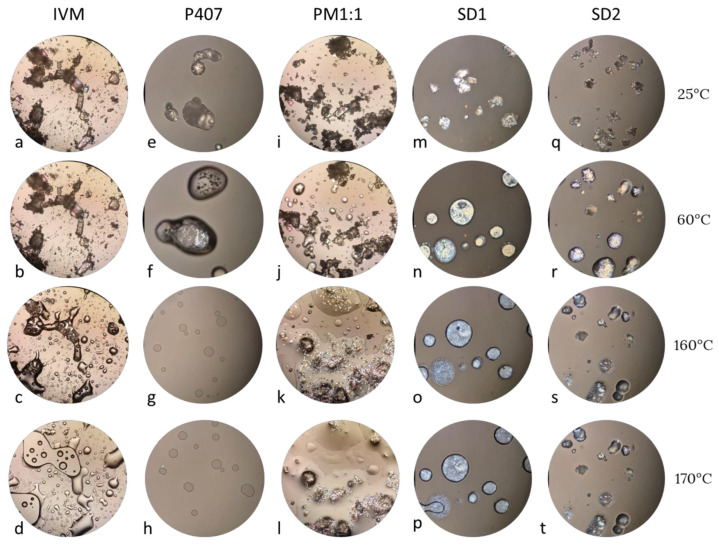
Hot-stage microscopy photomicrographs at 25 °C, 60 °C, 160 °C, and 170 °C (from top to bottom) of (**a**–**d**) IVM, (**e**–**h**) P40, (**i**–**l**) PM1:1, (**m**–**p**) SD1, and (**q**–**t**) SD2.

Subsequently, hot stage microscopy was performed to investigate the thermal behavior and physical state of the samples under controlled heating. As shown in Figure 6, IVM crystals remained unchanged at low temperatures; however, at 160 °C, the melting temperature could be observed. In the PM1:1, SD1, and SD2 samples, the IVM crystals were clearly visible within the molten polymer matrix at 60 °C, following the complete melting of the P407 polymer. In the PM1:1, the polymer appeared dispersed on the surface; in the SDs, however, P407 melted to form discrete droplets. Upon further heating, the IVM progressively dissolved or melted within the molten polymer phase, especially in the SDs, where no residual crystalline structures were evident at higher temperatures. These findings support the absence of the characteristic endothermic peak of IVM in the DSC curves, suggesting a possible molecular dispersion of IVM within the polymer matrix.

### 3.4. Solid Dispersions Characterization After Being Dispersed in the Water

#### 3.4.1. DLS

To assess the effect of polymer concentration on particle size distribution, SD1, SD2, SD3, and SD4 were dispersed into distilled water and analyzed by number-based DLS data (Figure S1 in Supplementary Material). For comparative purposes, P407 alone and P407 subjected to a rapid cooling process at final temperatures of 0 and 8.4 °C, which were used for the SDs preparation, were also analyzed.

As shown in Table 5, the particle size of P407 micelles alone was 26 nm, consistent with a previous report [49]. No significant effect of the final temperature on the size of the micelles was observed for P407 subjected to the SD1 and SD2 synthesis conditions. In the case of SD formulations, particle sizes increased to approximately 32 nm. This larger particle size compared to P407 micelles could be attributed to the molecular dispersion of IVM within the carrier. In particular, a slight decrease in particle size was observed for SD3, which contained higher amounts of P407. These results are supported by previous reports, which showed that increasing the concentration of P407 decreases the particle size of cubosomes [50]. In contrast, slightly larger particle sizes were observed for SD2 and SD4, both obtained at a final processing temperature of 8.4 °C. In this context, it can be inferred that neither the proportion of P407 nor the final processing temperature had a strong impact on the particle size of the SDs.

In terms of polydispersity, SD1 and SD3, prepared at a lower final temperature (0 °C), showed slightly lower PDI values than SD2 and SD4, which were obtained at 8.4 °C. These results evidence moderately dispersed samples and suggest that a lower final processing temperature may contribute to a more homogeneous particle population. On the other hand, the ZP values (Figure S2 in Supplementary Material) ranged from 19 to 22.4 mV, indicating the physical stability of the formulations in a dispersed state, in comparison with P407 alone. This suggests a reasonable degree of electrostatic repulsion between particles in suspension, minimizing aggregation during short-term storage [51].

#### 3.4.2. TEM

**Figure 7:**
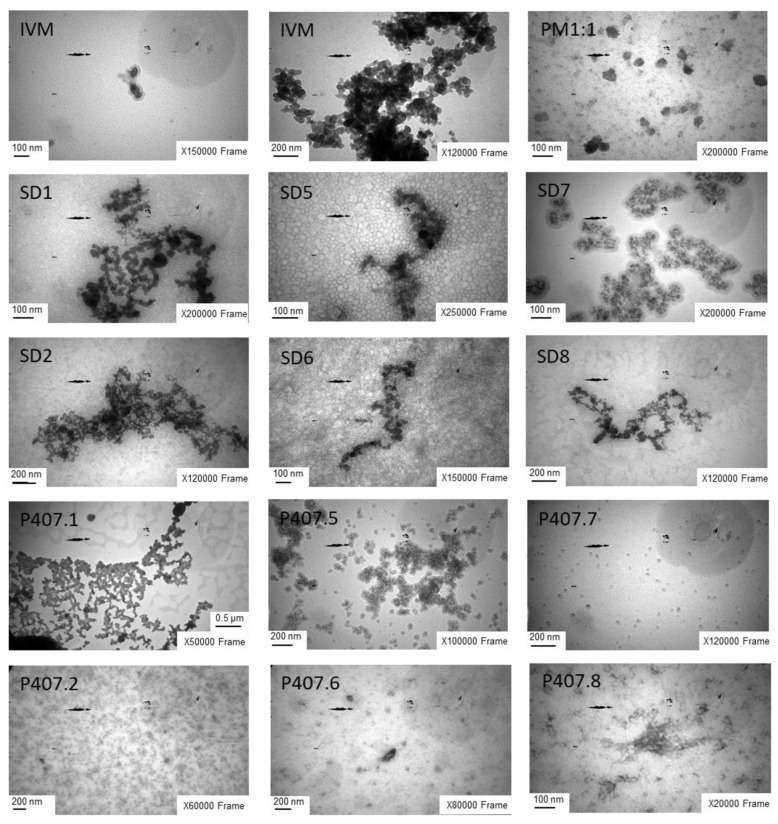
TEM images of IVM, PM1:1, and the P407 and SDs obtained under different synthesis conditions: SD1 and P407.1 (final temperature: 0 °C) and SD2 and P407.2 (final temperature: 8.4 °C) were prepared using a rapid cooling process; SD5 and P407.5 (final temperature: 0 °C) and SD6 and P407.6 (final temperature: 8.4 °C) using an intermediate cooling process; and SD7 and P407.7 (final temperature: 0 °C) and SD8 and P407.8 (final temperature: 8.4 °C) using a slow cooling process.

Taking into account the influence of temperature on the self-assembly properties of the P407 molecules, TEM, a powerful technique for structural characterization, was used to evaluate the impact of the synthesis conditions of SDs on their organization, morphology, and particle size. Figure 7 shows the images of IVM, PM1:1, SDs, and P407 samples suspended for comparative purposes, with P407 samples subjected to the same cooling gradient and final temperature used in the SDs preparation process.

IVM appeared as agglomerates of particles of 75 ± 9 nm, characterized by a darker black core. In contrast, PM1:1 showed the presence of both individual components, with particles measuring between 78 and 82 nm. In turn, P407 showed a spherical shape and smooth surface. However, P407 samples processed under identical SD conditions exhibited decreased agglomeration with the reduction of the cooling gradient, an effect that was particularly pronounced at a final temperature of 0 °C. As can be seen in the SD images, IVM appeared as a dark core enclosed by a lighter grey polymer matrix. The same influence of temperature on the P407 behavior was observed in each SD system. Interestingly, the particle size analysis indicated that the mean diameter of SD formulations was significantly lower than IVM. In particular, the SDs prepared under rapid cooling conditions exhibited larger agglomerates with particles of approximately 38 nm, attributable to a more disordered polymer self-assembly, which facilitates the entrapment of IVM within the P407 matrix. The particle size observed in the images is similar to that determined by DLS. In contrast, slower cooling conditions favored a more organized arrangement of polymer chains, resulting in larger particles (47–51 nm) and reduced IVM encapsulation, which resulted in a greater proportion of the drug remaining outside the polymeric network.

### 3.5. Studies in Solution

#### 3.5.1. Saturation Solubility Studies

Solubility is a crucial factor because it can affect drug absorption. To assess the impact of the polymer ratio, cooling gradient, and the final processing temperature used during SD preparation on the IVM solubilization, the saturation solubility was evaluated.

The solubility of IVM in SDs formulated with P407 excipient under different synthesis conditions, in both aqueous solution and SGF at 37.0 ± 0.1 °C, is summarized in Table 6. The data demonstrate the significant solubilization efficiency of all P407-based SDs compared to pure IVM. This enhancement in solubilization can be attributed to the IVM dispersion within the polymer matrix and the reduction in the crystallinity of both IVM and P407, as evidenced by XRPD analysis. These results are in agreement with previous reports [13,41].

The analysis of the solubility data revealed that SD1 and SD2 showed a higher increase in drug solubility than SD3 and SD4, suggesting that an increase in P407 content does not lead to proportional improvements in solubility. The nanometric sizes observed by DLS could explain the enhanced IVM solubility, whereas the similarity in particle sizes across formulations could potentially explain the comparable solubility values.

To evaluate the impact of the cooling gradient on IVM solubility, the results obtained using rapid (SD1 and SD2), intermediate (SD5 and SD6), and slow (SD7 and SD8) cooling ramps were compared. As shown in Table 6, for both final cooling temperatures (0 and 8.4 °C), the faster cooling rate resulted in greater IVM solubilization compared to the slower rates. Additionally, a lower final cooling temperature (0 °C) led to a slightly higher solubility. These findings can be attributed to differences in particle size, as determined by TEM. The SDs prepared under a rapid cooling rate were smaller than those prepared under intermediate or slow cooling conditions, which can explain the higher saturation solubility by the dispersion of IVM molecules within a more disordered P407 matrix.

#### 3.5.2. Dissolution Study

**Figure 8:**
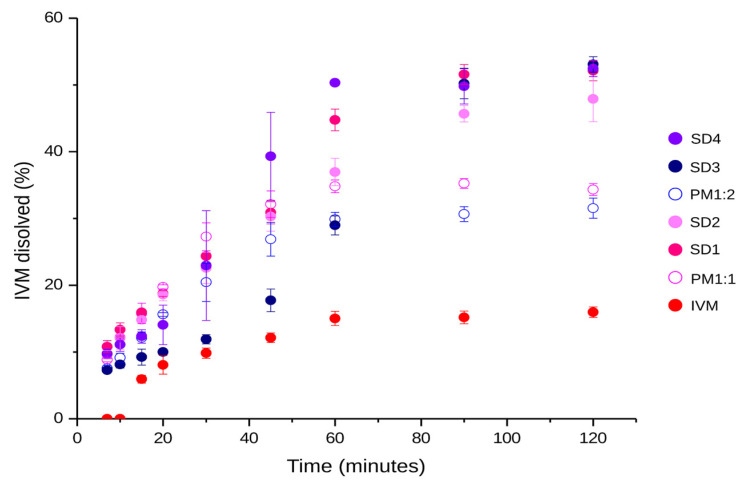
Dissolution profiles of IVM, PM1:1, PM1:2, SD1, SD2, SD3, and SD4. The data are expressed as mean ± standard deviation (n = 3).

According to the saturation solubility data, it was decided to assess the dissolution of SDs prepared using a rapid cooling process (SD1, SD2, SD3, and SD4). A significant difference in the percentage of dissolved IVM was observed between SDs, PMs, and the free drug. As illustrated in Figure 8, IVM, a poorly soluble drug, exhibited a reduced dissolution profile. In contrast, the SDs showed significantly enhanced IVM dissolution compared to the free drug and their respective PMs. These could be attributed to the reduction in particle size and decreased crystallinity, as evidenced by DLS and XRPD, respectively. This contributes to the polymer dissolution upon contact with the medium, facilitating the drug release and dissolution, in concordance with previous reports [13].

The dissolution percentages of SD1 and SD2 were found to be 53% and 49%, respectively, in contrast with 35% of the PM1:1. In a similar manner, SD3 and SD4 achieved significantly higher values of 54% and 53%, respectively, compared to the PM1:2, which showed a dissolved percentage of 31%. It was determined that a higher dissolution percentage was achieved for the SDs obtained at a final cooling temperature of 0 °C. However, in accordance with the results of solubility studies, it was observed that SD1 and SD3 achieved similar maximum dissolution percentages. This suggests that the dissolution of IVM in SGF does not increase with higher amounts of P407 in the system, as was reported in other studies [52]. This effect at higher polymer concentrations may be explained by the formation of a highly viscous barrier surrounding the solid particles, which limits solvent penetration and subsequently reduces the diffusion and dissolution rate of the drug.

Additionally, the dissolution profiles of SDs and PMs were evaluated in comparison with that of the reference drug (IVM) using the *f*2 factor (see Table 7). The calculated *f*2 values demonstrated that SD and PM profiles were non-similar to the free IVM, indicating that P407 markedly enhances the dissolution behavior of IVM.

### 3.6. In Vitro Drug Release Studies

**Figure 9:**
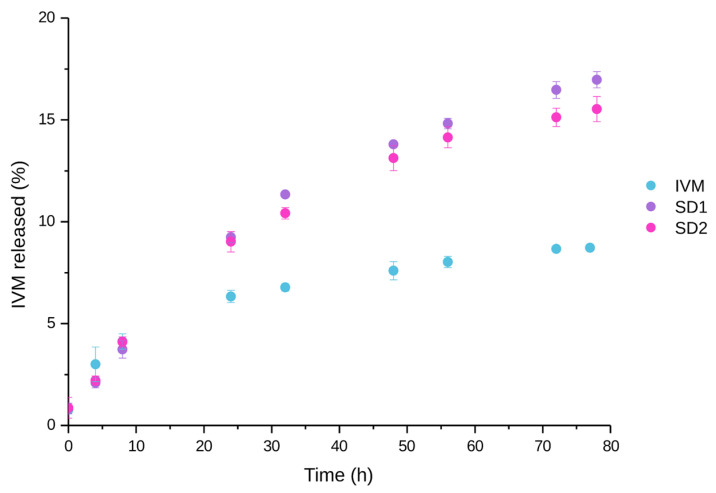
In vitro release of IVM from SD1 and SD2 compared to IVM at pH 7.4. The data are expressed as mean ± standard deviation (n = 3).

Finally, in vitro release studies were conducted using SD1 and SD2, selected as the optimized formulations based on their improved solubility and dissolution profiles, favorable thermal behavior, and efficient preparation conditions. The release of IVM from these formulations was evaluated in PBS (pH 7.4) by the dialysis bag method, and the results were compared with those of the pure IVM, as shown in Figure 9. The results demonstrate that SD1 and SD2 significantly increase the release of IVM compared to the free drug. In particular, the fastest release was observed in the case of SD1 and SD2 formulations. After 80 h, the cumulative %IVM released was 17% and 16% for SD1 and SD2, respectively, whereas pure IVM only achieved 9%. These differences can be attributed to the use of P407 as an excipient, which effectively improves IVM release. Notably, the final processing temperature did not appear to have a significant impact on the extent of release.

The release data obtained for SD1 and SD2 were fitted using different kinetic equations, including the zero-order, first-order, Higuchi, and Korsmeyer–Peppas models, to investigate the mechanism of the release process. Table 8 presents the transport constant (*k*) and correlation coefficient (r^2^) values obtained according to each model. The best fit for SDs was obtained with the Korsmeyer–Peppas model, indicating a good correlation within experimental data.

In the Korsmeyer–Peppas equation, the release exponent *n* incorporates information on the potential drug release mechanism from polymeric systems. The *n* values obtained for the release of IVM from SD1 and SD2 ranged between 0.45 and 0.89, indicating an anomalous (non-Fickian) drug release mechanism that was more pronounced in the case of SD1. The data revealed that the release process involves the simultaneous phenomena of diffusion through the polymer chains and relaxation of these chains [53,54].

## 4. Conclusions

In this study, P407-based SDs of IVM were successfully developed. Several characterization studies demonstrated good compatibility between the drug and the excipient, as well as a uniform distribution of the drug within the polymeric matrix. Among the formulations, SD1 and SD2, formulated under rapid cooling conditions, exhibited superior properties, including enhanced thermal stability, solubility, dissolution, and drug release compared to pure IVM. These findings successfully demonstrate the suitability of P407 to produce SDs using the fusion method. This approach represents a promising pharmaceutical strategy for improving the performance of IVM and increasing its biopharmaceutical properties. Consequently, P407-based SDs emerge as a valuable alternative formulation that provides a simple and scalable strategy to enhance the oral delivery of poorly soluble drugs and highlights the critical role of process parameters, such as the cooling gradient, in modulating the structural and functional characteristics of P407 in the final SD. Future studies could explore the in vivo performance of these optimized pharmaceutical systems, as well as the incorporation of additional functional excipients or targeting strategies to further expand their therapeutic potential.

## Figures and Tables

**Table 1:** Equations of the kinetic models.

Kinetic Model	Equation
Zero-order	*Q* = *k*_0_ × *t*
First-order	*ln*(100 − *Q*) = *ln Q*_0_ − *k*_1_ × *t*
Higuchi	*Q* = *k_H_* × *t*^1/2^
Korsemyer–Peppas	*Q* = *k_P_* × *t^n^*

**Table 2:** IVM solubility in aqueous solution containing polymeric water-soluble excipients at 37 °C.

Polymeric Excipient	Percentage (*w*/*v*)	IVM Solubility (µg/mL)	Solubility Increased (S/S_0_)
PEG6000	1	0.8 ± 0.2	0.3
	2	2.4 ± 0.5	0.9
	5	4.3 ± 0.2	1.7
PEG8000	1	2.7 ± 0.2	1.0
	2	1.9 ± 0.3	0.7
	5	5 ± 1	1.9
PVP-k30	1	25 ± 4	9.5
	2	32 ± 3	12.4
	5	99 ± 5	38.0
PVP-k90	1	4.2 ± 0.3	1.6
	2	20.0 ± 0.4	7.7
	5	9.3 ± 0.9	3.6
P188	1	16 ± 1	6.2
	2	14 ± 3	5.5
	5	30 ± 1	11.7
P407	1	(22 ± 1) × 10^2^	833.8
	2	(73 ± 1) × 10^1^	282.3
	5	147 ± 2	56.5
Sorbitol	1	12.0 ± 0.1	4.6
	2	3.4 ± 0.5	1.3
	5	1.6 ± 0.5	0.6

S_0_: solubility of the free drug.

**Table 3:** Synthesis conditions of SDs and their IVM content.

	Drug:P407 (*w*/*w*)	Cooling Ramp	Final Temperature (°C)	Drug Content (%)
SD1	1:1	Rapid	0	99 ± 1
SD2	1:1	Rapid	8.4	100.1 ± 0.3
SD3	1:2	Rapid	0	99.5 ± 0.1
SD4	1:2	Rapid	8.4	100.3 ± 0.8
SD5	1:1	Intermediate	0	100 ± 1
SD6	1:1	Intermediate	8.4	100.9 ± 0.4
SD7	1:1	Slow	0	98.8 ± 0.2
SD8	1:1	Slow	8.4	99.7 ± 0.7

**Table 4:** Crystallinity index (CI) and crystallite size of IVM, P407, PMs, and SDs.

Samples	IVM		P407	
	CI (%)	Crystallite Size (nm)	CI (%)	Crystallite Size (nm)
IVM	29.0	57.9	-	-
P407	-	-	62.4	22.0
PM1:1	16.1	57.3	24.3	23.3
PM1:2	5.3	57.4	36.6	20.0
SD1	5.2	44.5	29.3	20.1
SD2	5.3	50.0	30.3	20.5
SD3	5.2	43.4	39.5	21.6
SD4	5.3	51.6	36.5	21.4
SD5	5.6	50.2	32.8	20.9
SD6	5.4	51.6	33.1	21.6
SD7	5.3	56.3	33.5	21.8
SD8	6.0	56.6	33.9	22.4

**Table 5:** Particle size, PDI, and PZ of P407 and SDs.

Sample	Size (nm)	PDI	ZP
P407	26 ± 1	0.39 ± 0.04	5.7 ± 0.5
P407.1	25 ± 1	0.34 ± 0.03	5.5 ± 0.7
P407.2	25.2 ± 0.9	0.34 ± 0.05	5.1 ± 0.7
SD1	32 ± 1	0.37 ± 0.03	22.1 ± 0.4
SD2	32.9 ± 0.5	0.46 ± 0.04	19 ± 1
SD3	31.7 ± 0.8	0.37 ± 0.05	22.4 ± 0.9
SD4	32.2 ± 0.1	0.43 ± 0.05	21 ± 1

P407.1 (final temperature: 0 °C), P407.2 (final temperature: 8.4 °C).

**Table 6:** Saturation solubility of SDs in aqueous and SGF solutions.

Medium	H_2_O		SGF	
Sample	Solubility (µg/mL)	Solubility Increased (S/S_0_)	Solubility (µg/mL)	Solubility Increased (S/S_0_)
IVM	2.6 ± 0.4	-	4.80 ± 0.02	-
PM1:1	4671 ± 53	1797	6521 ± 553	1359
PM1:2	3619 ± 553	1392	6351 ± 724	1323
SD1	4207 ± 54	1618	5267 ± 156	1097
SD2	4135 ± 404	1590	5153 ± 98	1074
SD3	4055 ± 563	1560	4120 ± 243	858
SD4	3482 ± 433	1399	4203 ± 85	876
SD5	1450 ± 190	558	5210 ± 105	1085
SD6	602 ± 48	232	5052 ± 101	1053
SD7	529 ± 40	203	4983 ± 256	1038
SD8	490 ± 75	188	4296 ± 177	895

S_0_: solubility of the free drug.

**Table 7:** Percentage of IVM dissolved and *f*2 values.

Sample	Percentage of Dissolution	*f*2
IVM	15.0 ± 0.8	-
PM1:1	35.5 ± 0.8	40.1
PM1:2	31 ± 1	46.7
SD1	53 ± 2	32.5
SD2	49 ± 5	35.9
SD3	54 ± 2	37.2
SD4	53 ± 3	30.7

**Table 8:** Mathematical modeling of in vitro drug release using kinetic models.

Samples	Zero Order		First Order		Higuchi		Korsmeyer–Peppas		
	*k* (mg/h)	r^2^	*k* (mg/h)	r^2^	*k* (mg/h)	r^2^	*k* (mg/h)	r^2^	*n*
SD1	0.283	0.939	0.003	0.952	1.839	0.951	1.172	0.988	0.701
SD2	0.297	0.921	0.003	0.936	1.922	0.965	1.062	0.990	0.659

## Data Availability

All data are included in the article. Further inquiries can be directed to the corresponding author.

## Supplementary Materials

The following supporting information can be downloaded at: https://www.mdpi.com/article/10.3390/pharmaceutics17091101/s1, Figure S1: Particle size distribution by number; Figure S2: Z Potential.

## Abbreviations

The following abbreviations are used in this manuscript:

IVM	Ivermectin
P407	Poloxamer 407
P188	Poloxamer 188
PVP-k90	Polyvinylpyrrolidone k90
PVP-k30	Polyvinylpyrrolidone k30
PEG8000	Polyethylene glycol 8000
PEG6000	Polyethylene glycol 6000
PBS	Phosphate buffer solution
SDs	Solid dispersions
PM1:1	Physical mixture 1:1 *w*/*w*
PM1:2	Physical mixture 1:2 *w*/*w*
SGF	Simulated gastric fluid
XRPD	X-ray powder diffraction
FT-IR	Fourier-transform infrared spectroscopy
ATR	Attenuated total reflectance
SEM	Scanning electron microscopy
TEM	Transmission electron microscopy
DSC	Differential scanning calorimetry
TGA	Thermogravimetric analysis
DLS	Dynamic light scattering
PDI	Polydispersity index
ZP	Zeta potential
IC	Crystallinity index
