# Supplementary material for: Poloxamer-Based Biomaterial as a Pharmaceutical Strategy to Improve the Ivermectin Performance
Source: Pharmaceutics. 2025 Aug 23;17(9):1101. doi: 10.3390/pharmaceutics17091101 (PMC12472695; doi:10.3390/pharmaceutics17091101)
Supplement: Supplementary file 1 [file pharmaceutics-17-01101-s001.zip › pharmaceutics-3825142-supplementary.pdf]

SUPPLEMENTARY MATERIAL

Poloxamer-based biomaterial as a pharmaceutical strategy to improve the Ivermectin performance

Belén Alejandra Mezzano[1], María Soledad Bueno[1], Valeria Cintia Fuertes[2], Marcela Raquel Longhi[1] and Claudia Garnero[1*]

[1] Departamento de Ciencias Farmacéuticas, Facultad de Ciencias Químicas, Universidad Nacional de Córdoba, Unidad de Investigación y Desarrollo en Tecnología Farmacéutica (UNITEFA) CONICET-UNC, Córdoba, Argentina.
[2] Departamento de Fisicoquímica, Facultad de Ciencias Químicas, Universidad Nacional de Córdoba, Instituto de Investigaciones en Fisicoquímica de Córdoba (INFIQC) CONICET-UNC, Córdoba, Argentina.
*Correspondence: cgarnero@unc.edu.ar (C.G.)

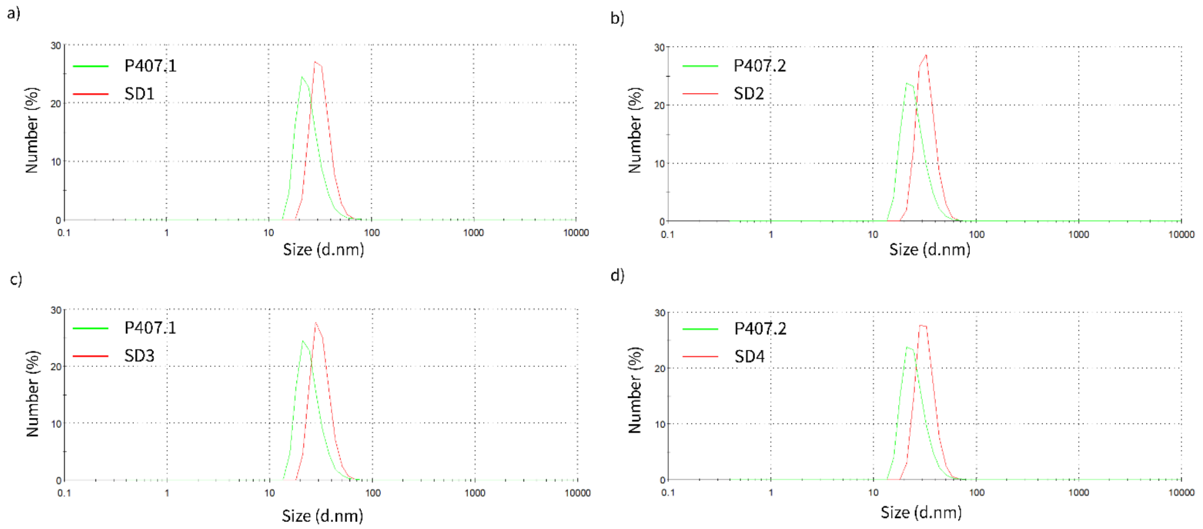

Figure S1. Particle size distribution by number of a) SD1 and c) SD3 in comparison with P407.1, b) SD2 and d) SD4 in comparison with P407.2, determined by DLS.

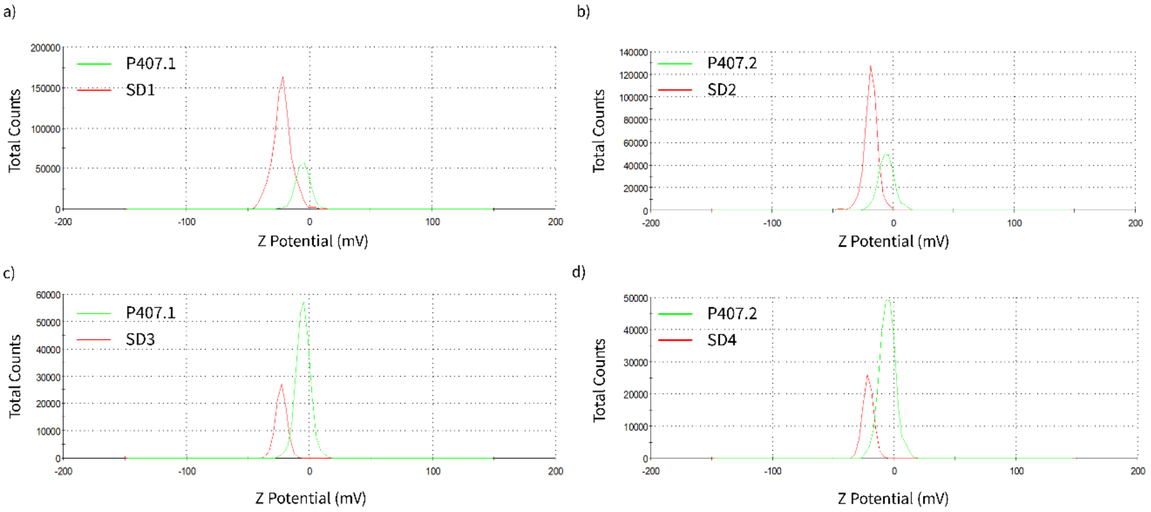

Figure S2. Z Potential of a) SD1 and c) SD3 in comparison with P407.1, b) SD2 and d) SD4 in comparison with P407.2, determined by DLS.
